# Zebrafish *mafbb* Mutants Display Osteoclast Over-Activation and Bone Deformity Resembling Osteolysis in MCTO Patients

**DOI:** 10.3390/biom11030480

**Published:** 2021-03-23

**Authors:** Yujie Han, Weihao Shao, Dan Zhong, Cui Ma, Xiaona Wei, Abrar Ahmed, Tingting Yu, Wei Jing, Lili Jing

**Affiliations:** 1 Engineering Research Center of Cell & Therapeutic Antibody, Ministry of Education, School of Pharmacy, Shanghai Jiao Tong University, Shanghai 200240, China; hanyujie@sjtu.edu.cn (Y.H.); shaoweihao1995@163.com (W.S.); maoer_zd@sjtu.edu.cn (D.Z.); ma-cui@sjtu.edu.cn (C.M.); weixiaona@sjtu.edu.cn (X.W.); Abrar22@sjtu.edu.cn (A.A.); 2 Shanghai Children’s Medical Center, Department of Medical Genetics and Molecular Diagnostic Laboratory, Shanghai Jiao Tong University School of Medicine, Shanghai 200127, China; ytt.007@163.com; 3 Department of Hepatobiliary Pancreatic Surgery, Shanghai Changhai Hospital, Shanghai 200433, China

**Keywords:** MAFB, Multicentric Carpotarsal Osteolysis (MCTO), osteoclasts, macrophage and monocytes, zebrafish

## Abstract

Multicentric carpotarsal osteolysis (MCTO) is a rare skeletal dysplasia with osteolysis at the carpal and tarsal bones. Heterozygous missense mutations in the transcription factor MAFB are found in patients with MCTO. MAFB is reported to negatively regulate osteoclastogenesis in vitro. However, the in vivo function of MAFB and its relation to MCTO remains unknown. In this study, we generated zebrafish MAFB homolog *mafbb* mutant utilizing CRISPR/Cas9 technology. *Mafbb* deficient zebrafish demonstrated enhanced osteoclast cell differentiation and abnormal cartilage and bone development resembling MCTO patients. It is known that osteoclasts are hematopoietic cells derived from macrophages. Loss of *mafbb* caused selective expansion of definitive macrophages and myeloid cells, supporting that *mafbb* restricts myeloid differentiation in vivo. We also demonstrate that MAFB MCTO mutations failed to rescue the defective osteoclastogenesis in *mafbb^−/−^* embryos, but did not affect osteoclast cells in wild type embryos. The mechanism of MCTO mutations is likely haploinsufficiency. Zebrafish *mafbb* mutant provides a useful model to study the function of MAFB in osteoclastogenesis and the related MCTO disease.

## 1. Introduction

The bZip factor MafB (v-maf musculoaponeurotic fibrosarcoma oncogene ortholog B) is a member of the large Maf transcription factor family [1]. It is expressed in multiple tissues including the spinal cord, retina and hematopoietic cells, and plays diverse functions in tissue development and cellular differentiation [1,2,3,4,5]. MafB is highly expressed in monocytes and macrophages, and is important for macrophage differentiation [1,4,6]. It has also been demonstrated that MafB regulates osteoclastogenesis [7].

Osteoclasts are bone-resorbing cells that are derived from the hematopoietic monocyte-macrophage lineage [8,9]. They are located at or near the bone surface, and degrade the bone in a specialized extracellular compartment called Howship’s lacunae by secreting acid and lytic enzymes, like tartrate-resistant acid phosphatase (TRAcP) and Cathepsin K(CTSK) [10]. Osteoclast cells are indispensable for normal bone development and remodeling [11].

The formation of osteoclast cells is regulated by a series of signaling molecules. Two main factors, macrophage colony stimulating factor (M-CSF) and receptor activator of NF-κB ligand (RANKL) positively regulate the osteoclast differentiation [12]. After binding to their respective receptors (M-CSFR or RANK) on the cell surface, they are able to induce the expression of nuclear factor of activated T-cells cytoplasmic 1 (NFATc1)—the master regulator of osteoclastogenesis [7]. Osteoprotegerin (OPG) is an important inhibiting factor of osteoclastogenesis by blocking RANKL binding to RANK [13]. It is known that MafB inhibits the expression of NFATc1 and the osteoclast-associated receptor (OSCAR) during RANKL-mediated osteoclastogenesis, and thereby negatively regulates osteoclastogenesis in vitro [7]. However, the in vivo function of MafB in osteoclastogenesis has not been demonstrated.

Mutations in MAFB have been reported to be responsible for the multicentric carpotarsal osteolysis syndrome (MCTO) [14,15,16]. MCTO is a rare skeletal and nephropathic disorder, and the responsible mutations are all missense mutations in the amino-terminal transactivation domain of MAFB. The patients show aggressive osteolysis, predominantly in the carpal and tarsal bones [17,18]. It is hypothesized that the patients might have overproduced osteoclasts, which eventually leads to underdeveloped and deformed bone formation. However, how MCTO mutation leads to the disease pathogenesis remains unknown. Understanding the function and mechanism of MAFB in normal and pathological conditions will help identify novel therapeutic targets.

Animal models are powerful tools to study the function of genetic mutations involved in disease development. MafB null mutant (*MafB*^−/−^) mice and mice containing an MCTO mutation (*MafB*^MCTO/MCTO^) have been generated [6,19]. These mice show nephropathic symptoms of glomerular sclerosis which is similar to MCTO patients. However, the osteoclast formation and bone development defects in these mice have not been reported. As an alternative animal model, zebrafish has recently been used to study bone development and bone disease. The osteoclast cells and skeletal physiology are similar between zebrafish and mammals [20,21,22]. The biochemical networks and metabolic pathways in zebrafish and mammals are also largely conserved. Moreover, zebrafish embryos are relatively transparent, and the osteoclast cell and bone development can be easily observed by real-time live imaging [23].

There are two paralogs of human MAFB in zebrafish, *mafba* and *mafbb*, and *mafbb* is more preferentially expressed in myeloid lineages during embryogenesis [24,25,26]. We generated *mafbb* mutants by CRISPR-Cas9 technology, and characterized osteoclast and bone development in the mutants. We then tested the function of MAFB MCTO mutations in *mafbb*^−/−^ embryos. Our results demonstrated that MAFB negatively regulates osteoclast differentiation in vivo and MCTO mutation is a loss-of-function of MAFB. Zebrafish *mafbb* mutant provides a useful model to study the in vivo function of MafB and the MCTO disease.

## 2. Materials and Methods

### 2.1. Zebrafish Maintenance and Embryo Handling

The wild type (WT) AB and transgenic zebrafish were maintained, handled, and bred according to standard protocols from the Institutional Animal Care Committee of Shanghai Jiao Tong University. Adult zebrafish were raised in a circulating water system under a 14 h/10 h light/dark cycle at 26–28 °C and fed two times per day. Adult male and female zebrafish were kept separately in the same mating box in the evening and mated the following morning. The embryos were collected and kept at 28.5 °C in E3 medium (5 mM NaCl, 0.17 mM KCl, 0.33 mM CaCl_2_, and 0.33 mM MgSO_4_) with a density of 100 embryos per 10-cm-diameter Petri dish. Embryos were staged by hours post-fertilization (hpf) and days post-fertilization (dpf).

### 2.2. Generation and Analysis of Tg(mafbb:GFP) and Tg(ctsk:mGFP)

To generate Tg(*mafbb*:GFP), the plasmid (pT2-cryR; *mafbb*CE1-P1Egfp, from Addgene, 20 ng/μL) and 30 ng/μL Tol2 mRNA were co-injected into WT embryos at the one-cell stage. The embryos showing a positive expression of GFP were raised to adults (F0 founder). F0 fish were outcrossed to WT fish, and GFP positive embryos were raised to adults (F1). F1 fish were outcrossed WT again to obtain stable transgenic lines. Transgenic lines were established from two different F0 founders.

The plasmid (*ctsk*:mGFP) was generously provided by Dr. C. Winkler from National University of Singapore. The plasmid (20 ng/μL) with 10 ng/μL I-SceI (#R0694S, NEB, Ipswitch, MA, USA) was co-injected into WT embryos at the one-cell stage. The stable Tg(*ctsk*:mGFP) line was generated similar to Tg(*mafbb*:GFP).

For analysis, the embryos were mounted in 1% low-melt agarose and imaged under a confocal microscope Leica SP8 microsystems (Leica, Heidelberg, DE, Germany).

### 2.3. Generation of mafbb Knockout Mutants

**Figure 1:**
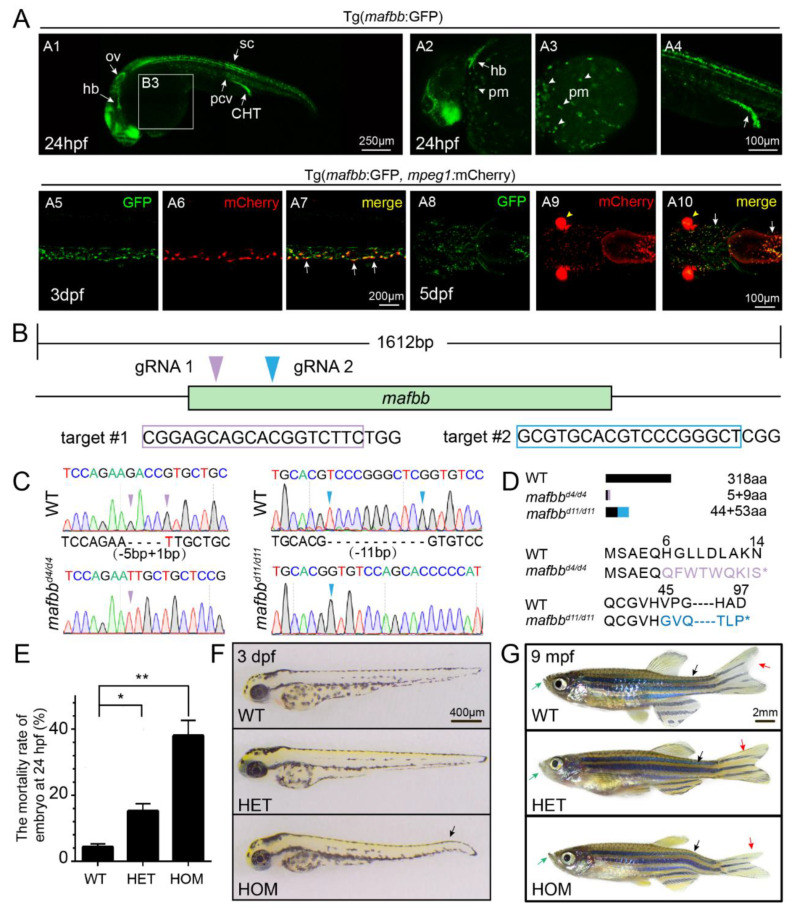
The expression of *mafbb* during embryogenesis and generation of *mafbb* mutants. (**A**) Tg (*mafbb*:GFP) shows *mafbb* expression in primitive myeloid (pm) cells, hindbrain (hb), otic vesicle (ov), spinal cord (sc), posterior cardinal vein (pcv), caudal hematopoietic tail region (CHT) (A1–4). A2–4 are enlarged images of A1. The white arrowheads in A2 and A3 point to pm cells. The double Tg(*mafbb*:GFP;*mpeg1*:mCherry) shows the expression of GFP in mCherry^+^-macrophages in the CHT (A5–7, lateral views) and in the head region (A8–10, ventral views). The white arrows in (A7 and A10) point to the overlapped expression of *mafbb* and *mepg1*. The arrowheads in (A9–10) mark the eyes from α-crystallin-mCherry in the plasmid of *mabb*:GFP [32]. (**B**) The schematic diagram of *mafbb* cDNA and the targeted regions of two guide RNAs. The target DNA sequences are shown in purple or blue rectangles. (**C**) Sanger sequencing analysis of PCR fragments containing gRNA1 and gRNA2 targeted regions from *mafbb* deficient homozygotes. The deleted nucleotides are replaced by -, and the inserted nucleotides are in red. (**D**) Schematic representation and amino acid sequence of the wild type MafBb and two predicted truncated proteins. (**E**) The mortality rate of embryos at 24 hpf (n = 315–688 per group). Results are expressed as mean ± SEM, (* *p* < 0.05, ** *p* < 0.01, *t* test). The statistical significance was displayed as (**F**) Images of embryos at 3 dpf. The black arrow points to the curved tail. (**G**) Images of adult zebrafish at 9 mpf. The projecting lower jaw (green arrows), the curved spine (black arrows) and the asymmetric caudal fin (red arrows) are shown in *mafbb*^−/−^ mutants. WT, wild type; HET, *mafbb^d^*^11*/+*^; HOM, *mafbb^d^*^11*/d*11^; mpf, month post fertilization.

*mafbb* knockout mutants were generated through the CRISPR-Cas9 system and performed following the protocol as described in [27]. Two *mafbb* specific guided RNAs (as in Figure 1B) were designed to target the beginning site of the exon. One-cell stage WT embryos were injected with 1 nL of the solution containing 100 ng/µL Cas9 mRNA and 20 ng/µL gRNA. Injected F0 fish were grown to adulthood and outcrossed to WT fish. F1 mutant offsprings were identified with T7 endonuclease I (T7E1) assay (M0302S, NEB, Ipswitch, MA, USA) using primers around the target loci. Each target loci was amplified by PCR from the genomic DNA and the mutation was revealed by DNA sequencing. F1 fish were outcrossed to WT to obtain stable F2 mutant lines. Primers used in T7E1 assay and PCR amplification are listed in Table A1 (Appendix A).

### 2.4. Synthesis of Antisense Probes and Whole-Mount In Situ Hybridization (WISH)

We searched the NCBI genebank for sequences of *ctsk* (Cathepsin K) (Source: ZFIN; Acc: ZDB-GENE-001205-4) and *rank/tnfrsf1b* (tumor necrosis factor receptor superfamily, member 1B (Source: ZFIN; Acc: ZDB-GENE-070410-133). Briefly, the total RNA extracted from 6 dpf AB larvae was reverse transcribed to cDNA. The cDNA was then used to get a 1 kb fragment of *ctsk/rank* through PCR. The PCR products were cloned into the Ecor1/Xbal site of the pCS2 (+) backbone vector. SP6 RNA-polymerase (P1088, Promega, Madison, WI, USA) was used to synthesize the digoxigenin-labeled RNA probes. PCR primer sequences are from the literature [28] and listed in Table A2. The PCR products were confirmed by sequencing.

Whole-mount in situ hybridization was performed using digoxigenin-UTP labeled RNA probes (*pu.1, runx1; cmyb; mpx; rag1; hbbe1; mfap4; apoe; ctsk; rank*). Embryos at the desired time point were fixed overnight in 4% paraformaldehyde (PFA) at 4 °C, bleached and dehydrated in methanol at −20 °C for at least two hours. Further processing of embryos was conducted according to the previous protocol [29]. The stained embryos were imaged under SZX16 stereomicroscope or BX53 microscope (Olympus, Tokyo, Japan).

### 2.5. Neutral Red Staining and Benzidine Staining

Optimal staining of macrophages in live embryos was obtained by incubating 3 dpf or 5 dpf embryos in 2.5 μg/mL neutral red/E3 medium (A600652, Sangon Biotech, Shanghai, China) at 28.5 °C in the dark for 6–8 h according to the protocol in [30]. Benzidine staining was done according to [31] with some modification. Larvae were incubated in benzidine (B108444, Aladdin, Shanghai, China) staining solution (2 mL 5 mg/mL benzidine/methanol, 16.7 μL 3 M NaOAc solution, 100 μL H_2_O_2_ and 2.483 mL H_2_O) for 30 min in dark, then washed by PBT and fixed in 4%PFA overnight. The stained embryos were imaged under SZX16 stereomicroscope or BX53 microscope (Olympus, Tokyo, Japan).

### 2.6. Tartrate-Resistant Acid Phosphatase (TRAcP) Staining

TRAcP staining on zebrafish and zebrafish scales was performed as described previously by using ACID PHOSPHATASE, LEUKOCYTE (Procedure No. 387, Sigma-Aldrich, St Louis, MO, USA) with modification [32]. Briefly, adult zebrafish were fixed in 4% PFA at 4 °C overnight. The fish were then eviscerated to collect the scales for TRAcP staining. For some fish, the rest parts were used to do alizarin red staining. For TRAcP staining on 2 mpf zebrafish, the muscular tissue near the spine was removed first with blades and forceps after fixation.

**Figure 2:**
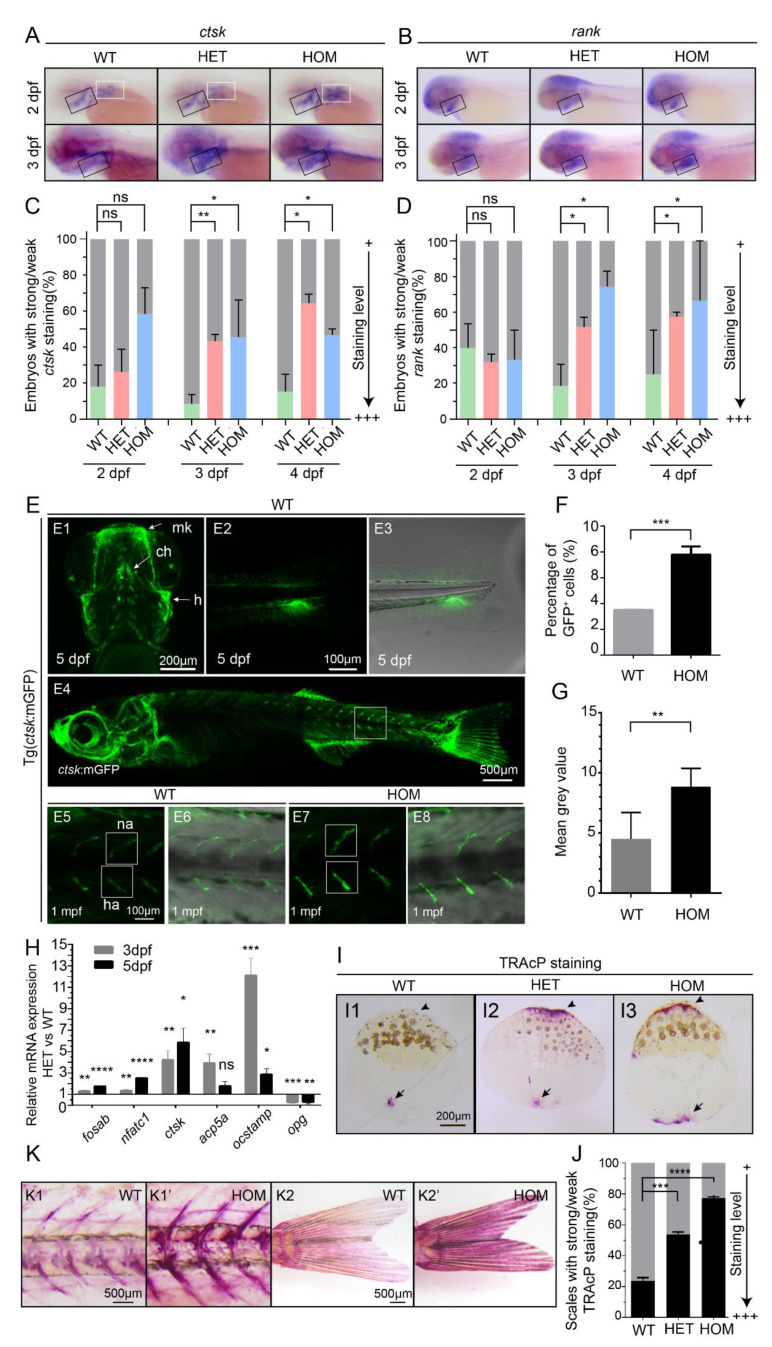
Overproduction of osteoclast cells in *mafbb* mutants. (**A**,**B**) WISH of *ctsk* and *rank* in embryos at 2–3 dpf. Black boxes indicate the pharyngeal arches; white boxes indicate the pectoral fins. (**C**,**D**) Quantification of the *ctsk* and *rank* expression during the early stages of development. 30–50 embryos were examined for each group. (**E**) Fluorescent images of Tg(*ctsk*:mGFP) at 5 dpf (E1–3) and 1 mpf (E4–6) in WT embryos, and at 1 mpf in HOM embryos (E7,8). E1, the ventral view of the head; Meckel’s cartilage (mk), ceratohyal (ch), hyosymplectic (h); E2, the lateral view of the tail; E3, overlay of E2 and transmitted light brightfield image; E4, the lateral view of 1 mpf Tg (*ctsk*:mGFP); E5–8, higher magnifications of boxed area in E4, neural arch (na); hemal arch (ha); E6 and E8, overlay of fluorescence and transmitted light brightfield image; E5,6, WT; E7,8, HOM. (**F**) Quantification of *ctsk*-GFP^+^cells in embryos by flow cytometry in Tg (*ctsk*:GFP) embryos at 5 dpf. (**G**) Mean grey value of GFP fluorescence intensity in the white boxes in E5 and E7. 3 fish were analyzed for each group. (**H**) Relative expression of genes involved osteoclast development (*fosab; nfatc1; ctsk; acp5a; ocstamp; opg*) in *ctsk*-GFP^+^ cells sorted from WT and HET embryos at 3–5 dpf. (**I**) Representative images of TRAcP histochemical staining on scales from adult zebrafish. The arrows and arrowheads point to the increased TRAcP activity, and the arrowheads point to the scale border. (**J**) Summary of the scales from different adult zebrafishes with TRAcP staining (n = 296–488 scales from 3 zebrafish per group; weak staining as in I1, strong staining as in I2,3 with increased TRAcP coloring along the scale border). (**K**) TRAcP staining of 2 mpf zebrafish. Vertebral columns (K1&K1′) and caudal fins (K2&K2′). 3 zebrafish were examined for each group with a consistent phenotype. WT, wild type; HET, *mafbb^d^*^11*/+*^; HOM, *mafbb^d^*^11*/d*11^. Results in C, F, G, H and J are expressed as mean ± SEM, (* *p* < 0.05, ** *p* < 0.01, *** *p* < 0.001, **** *p* < 0.0001, *t* test, ns, not significant).

Scales or whole zebrafish were preprocessed for 2 h at room temperature in tartrate buffer [0.2 M acetate buffer (pH 5.2) with 50 mM L(+)-Tartrate buffer (pH 4.9)] and then incubated in TRAcP staining solution (70 μg/mL Fast Garnet GBC Base Solution; 1 mM Sodium Nitrite Solution; 125 μg/mL Naphthol AS-BI Phosphoric Acid Solution; 0.1 M Acetate Solution; 6.7 Mm L(+)-Tartrate buffer) in the dark for 2 h. The samples were washed three times by PBT before taking images. For quantification of scale TRAcP staining, weak staining means little or no staining at the scale border as in Figure 2I1, and strong staining means a large area of staining as in I2-3 or more at the scale border.

### 2.7. MicroCT Scans

10-mpf (months post-fertilization) were fixed in 4% PFA O/N, then stored in ethanol and scanned with a Skyscan 1176 microCT system (Bruker, Billerica, MA, USA) at 45 kV and 450 μA. We used a voxel size of 18um as resolution. The 3D evaluation was conducted using CTAn (Bruker, Billerica, MA, USA). Quantification of bone morphology was performed on the hypural bone next to the caudal fin, similar to the studies in [33,34]. Determined parameters were bone volume density (BV/TV) and the mean value of grey-level intensity which is corresponded to the relative bone density.

### 2.8. Alcian Blue Staining and Alizarin Red Staining

Cartilage was stained by alcian blue (AB) solution (015-13805, Wako, Osaka, Japan) according to the protocol described in [35]. 5 dpf embryos were fixed in 4% PFA at 4 °C overnight and bleached in 1% KOH/3% H_2_O_2_ solution until the pigments were cleared. After that, embryos were incubated with acid alcohol (1% HCl in 70% ethanol) for 20 min before transferred into a 0.1% alcian blue in acid alcohol for 2 h. The specimens were mounted in 70% glycerol and photographed after washed three times, 30 min per time by acid alcohol.

For larvae teeth staining, 8 dpf larvae were fixed and bleached as for alizarin red staining, following the procedure in [35]. The larvae were incubated with 1 mg/mL Alizarin red S (71001954, China National Medicines Corporation Ltd., Beijing, China) in 1% KOH for 1 h and digested for several hours in 1 mg/mL trypsin (A100260-0250, Sangon Biotech, Shanghai, China) in 2% borax (10020808, China National Medicines Corporation Ltd., Beijing, China).

Adult zebrafish bone staining was similar to larval bone staining. Briefly, 9-month old zebrafish were anesthetized with 0.02% tricaine and sacrificed on ice before fixed in 4% PFA at 4 °C overnight. The specimens were bleached for 1 day and transferred to 30 % saturated sodium tetraborate overnight. Afterwards, the specimens were placed in 1% KOH with 1 mg/mL Alizarin Red overnight and 1% trypsin and 2% borax were added until 85% of the soft tissue was dissolved. The specimens were transferred through a series of 1% KOH/glycerol solutions until they settled at the bottom. The specimens were transferred to 70% glycerol for long-term storage and photographing.

### 2.9. Whole Kidney Marrow Cell Collection and Cytology

The adult fish kidney marrow was dissected and placed into tubes containing 400 μL FBS solution. The single hematopoietic cells from kidney marrow were generated by pipetting and filtration through 40-µm filters. Single-cell suspensions were diluted to 15,000-30,000 cells/mL and cytocentrifuged at 400 rpm for 4 min with cytospin 4 (Sigma-Aldrich, St Louis, MO, USA). Blood smears were processed by May-Grünwald and Giemsa double stain (63590/48900, Sigma-Aldrich, St Louis, MO, USA) for morphological analysis and differential cell counts.

### 2.10. Flow Cytometry and Cell Sorting and Counting

We sorted *ctsk^+^* cells from Tg (*ctsk*:mGFP) in WT and *mafbb^+^*^/−^ embryos at 3 dpf and 5dpf. Embryos were anesthetized with 0.02% tricaine and washed with sterile E3 solution. Single cells were collected by shredding larvae with a blade and incubated for 20 min (37 °C) with 38 μg/mL Liberase (05401119001, Roche, Basel, Switzerland). 10% FBS was added to stop the reaction, followed by filtration (40 µm filter) and centrifugation (5000 rpm, 4 °C, 15min). The supernatant was removed, and cells were resuspended with 800 µL 2% FBS. GFP negative and positive cells were sorted with FACS AriaII (Becton, Dickinson and Company, NJ, USA).

We counted *mpeg1^+^* cells from Tg (*mpeg1:*mCherry) in WT and *mafbb*^−/−^ embryos, and *ctsk^+^* cells from Tg (*ctsk:*mGFP) in WT and *mafbb*^−/−^ embryos at 5 dpf. The procedure was conducted as cell sorting. mCherry^+^ or GFP^+^ cells were counted using LSRFortessa (Becton, Dickinson and Company, Franklin lakes, NJ, USA).

### 2.11. Gene Expression by Real-Time qPCR

Gene expression was evaluated using real-time qPCR. Briefly, total RNA was extracted from embryos with TRIzol reagent (10296028, Thermo Fisher Scientific, Waltham, MA, USA). cDNAs were synthesized from total RNA using the PrimeScript^TM^RT reagent Kit with gDNA Eraser (RR047A, Takara, Shiga, Japan). TB Green Premix Ex Taq^TM^ ii (RR820A, Takara, Shiga, Japan) was used for qPCR analysis. Each target gene was calculated using the 2^−ΔΔCT^ method [36]. The primers for different target genes and β-actin (the reference gene) are listed in Table A3.

### 2.12. Alendronate Treatment

24-hpf embryos were continuously exposed to 100 μM Alendronate (129318-43-0, Absin, Shanghai, China) for 2-4 days [37]. The control groups were treated with DMSO. 3-dpf embryos were used for WISH *(ctsk)*. 5-dpf embryos were used for AB staining and confocal imaging.

### 2.13. mRNA Injection

Human MAFB^WT^, MAFB^P59L^, MAFB^P63R^, MAFB^S70L^ cDNA was separately cloned to the PCS2(+) plasmid. mRNA was transcribed using mMESSAGE mMACHINE SP6 Transcription Kit (AM1340, Thermo Fisher Scientific, Waltham, MA, USA). 1 nL of WT or mutant MAFB mRNA (50 ng/μL) was, respectively, injected into WT or *mafbb^−/−^* embryos at one-cell stage.

### 2.14. Statistical Analysis

GraphPad Prism 8.0.2 (GraphPad Software, San Diego, CA, USA, 2019, https://www.graphpad.com, accessed on 21 March 2021) was used to analyze all data. The values of all triplicate experiments are presented as mean ± SEM. The statistical significance was displayed as “ns” for no statistical significance, “*” for *p* < 0.05, “**” for *p* < 0.01, “***” for *p* < 0.001, and “****” for *p* < 0.0001. The unpaired 2-tailed student *t*-test was used for data analysis. For statistical analysis with WISH and other staining results, the groups with strong stainings are used in *t*-test.

## 3. Results

### 3.1. Generation of Zebrafish mafbb Mutants Using the CRISPR/Cas9 System

Zebrafish contains two *mafb* genes, *mafba* and *mafbb*. The two genes are expressed in distinct and overlapping areas during development. Previous studies have suggested that *MafB* is highly expressed in myelomonocytic lineages of hematopoietic cells in mice [3]. In zebrafish, *mafbb* is highly expressed in myeloid lineages and macrophages, we therefore focused on *mafbb*. To further explore the expression of *mafbb* in zebrafish, we generated a transgenic line using the construct (*mafbb*CE1-P1E:GFP) in which an enhancer and a cognate promoter element of *mafbb* gene are used to drive the expression of the enhanced green fluorescent protein [38]. At 24 hpf, transgenic embryos showed good GFP expression in primitive myeloid (pm) cells, the caudal hematopoietic tissue (CHT), posterior cardinal vein (pcv), hindbrain (hb) and otic vesicle (ot) during the early stages of development (Figure 1A,A1–4), similar to the analysis from whole-mount in situ hybridization (WISH) in zebrafish embryos [24], and to the GFP expression in mouse transgene containing *MafB* 5′-upstream fragment fused to GFP [3]. Tg(*mafbb*:GFP) was crossed to Tg(*mpeg1*:mCherry) which directs mCherry expression in macrophages [39]. We observed that a large number of mCherry^+^ macrophages co-expressed GFP in the head and the CHT region at 3 and 5 dpf (Figure 1A,A5–10). These data support the robust expression of *mafbb* in macrophages, and indicate a potential function of *mafbb* in macrophage and osteoclast cells.

To study the role of *mafbb* in zebrafish osteoclast differentiation and bone development, we generated *mafbb* mutant utilizing CRISPR/Cas9 technology. The 318 aa protein Mafbb is encoded by the 1612 bp gene *mafbb*, which contains one exon (Figure 1B). A guide RNA that targets the N-terminal region of *mafbb* exon (Figure 1B) was designed and co-injected with Cas9 mRNA into one-cell stage WT embryos. After screening several founders that transmitted to F1 progeny, we established a stable line named *mafbb^d^*^11*/d*11^ that has a frameshift mutation caused by a deletion of 11 bp. We also used a second gRNA with a different target site and obtained a second mutant line named *mafbb^d^*^4*/d*4^ with a deletion of 4 bp (Figure 1C,D). The two *mafbb* mutant lines demonstrate the same phenotypes. Here, we show the results from *mafbb^d^*^11^ allele for the rest of the paper.

The mortality rates in *mafbb^+/−^* and *mafbb^−/−^* were 15% and 40%, respectively, at 24 hpf, which were significantly increased compared to wild type (WT) embryos (Figure 1E). At 3 dpf, 20–40% of *mafbb^−/−^* embryos exhibited abnormal tail bending (Figure 1F). For *mafbb^−/−^* embryos that survived to the adults, many of them showed morphological defects. Around 50% of the fish have the protruding lower jaw, 50% show asymmetric caudal fin and, 10% demonstrate curved spine. These defects may occur together or independently. In contrast, WT and *mafbb^+/−^* zebrafish rarely showed similar abnormalities at the stages examined (Figure 1F,G).

### 3.2. Osteoclast Cell Development Is Enhanced in mafbb Deficient Embryos

To investigate whether *mafbb* is involved in osteoclastogenesis, we first examined the osteoclast-like cells in zebrafish embryos through WISH of two osteoclast makers, *ctsk* and *rank* [29]. The WISH results showed that *ctsk* (Figure 2A) and *rank* (Figure 2B) were expressed in the pharyngeal arches and the pectoral fin, as in the previous report [28]. At 2 dpf, the expression of *ctsk* and *rank* in *mafbb^+/−^* and *mafbb^−/−^* showed no difference compared to WT. However, at 3 and 4 dpf, the expression of *ctsk* and *rank* increased significantly in *mafbb^+/−^* and *mafbb^−/−^* (Figure 2C,D).

To analyze the osteoclast formation through the development course of *mafbb* mutants, we generated zebrafish Tg (*ctsk*:mGFP) using the plasmid expressing membrane bound EGFP (mEGFP) under the control of a medaka cathepsin K (*ctsk*) promoter [40]. Stable transgenic fish showed GFP expression comparable to the medaka transgenic line from the same plasmid [40], and to the zebrafish bacterial artificial chromosome (BAC) recombineering-based YFP labeled *ctsk* transgenic line [28]. At 5 dpf, GFP is expressed in the head (including the pharyngeal arches) and the tail region (likely in the caudal fin) (Figure 2E,E1–3). At this stage, GFP expression was increased in *mafbb^−/−^* embryos compared to WT siblings. The percentage of GFP-expressing osteoclast-like cells in *mafbb^−/−^* was significantly expanded in comparison to WT (7.8 ± 0.4% vs. 3.5± 0.01%, Figure 2F). At later stages, GFP expression was found in the vertebrate column, around the neural and haemal arches (Figure 2E4), similar to the expression shown in [40]. In 1-month-old *mafbb^−/−^* zebrafish, GFP expression along the neural and haemal arches was strongly increased compared to WT siblings (Figure 2E8,G).

To further analyze the effects of *mafbb* on osteoclast differentiation, we sorted *ctsk*^+^ cells from Tg (*ctsk*:mGFP) in WT and *mafbb*^+/−^ embryos, and examined the relevant gene expression related to osteoclastogenesis by RT-PCR (Figure 2H). The results showed that osteoclast differentiation genes *fosab* and *nfatc1*, osteoclast maturation genes *ctsk, acp5a* and *ocstamp* [41] were all upregulated in *ctsk*^+^ cells sorted from *mafbb*^+/−^ compared to WT at 3 and 5 dpf. However, the inhibitor factor *opg* (also known as *tnfrsf11b*) [13] was downregulated in *mafbb*^+/−^ at 3 and 5 dpf (Figure 2H). These data support that osteoclast differentiation is enhanced when *mafbb* is down-regulated.

To evaluate osteoclast cells in adults, we used TRAcP enzyme staining on adult zebrafish scales, which are part of the dermal skeleton and contain both osteoblasts and osteoclasts. The results showed significant enhancement of TRAcP activity in *mafbb^+/−^* and *mafbb^−/−^* scales, particularly at the scale border compared to the WT (Figure 2I,J). We also performed TRAcP staining on adult zebrafish, and found enhanced osteoclast activity in the spine and caudal fin of *mafbb^−/−^* fish compared to WT siblings (Figure 2K). Thus, the development and maturation of osteoclast cells are persistently increased in *mafbb*-deficient zebrafish.

### 3.3. mafbb Deficiency Results in Abnormal Cartilage and Bone Development

**Figure 3:**
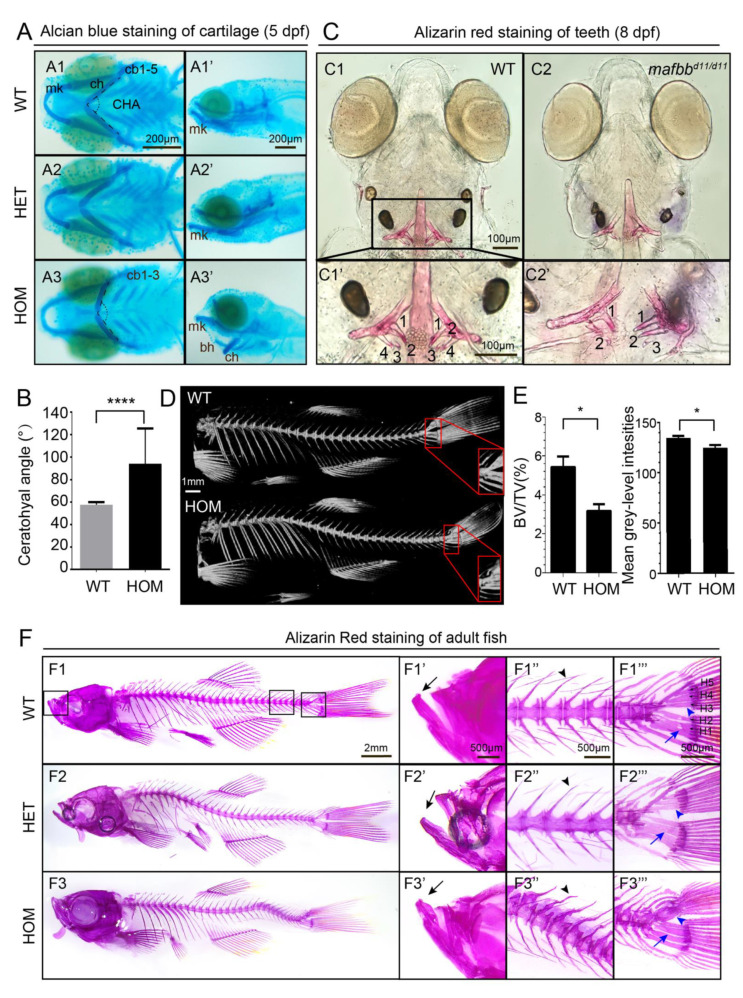
Abnormal cartilage and bone formation in *mafbb* mutants. (**A**) Alcian Blue staining of embryos at 5 dpf. A1-A3, ventral views; A1′-A3′, lateral views. Ceratobranchial pairs (cb); ceratohyal (ch); Meckel’s cartilage (mk); basihyal (bh), CHA (ceratohyal angel). (**B**) Summary of CHA in embryos at 5 dpf (n = 15 embryos per group). (**C**) Ventral views of Alizarin Red (AR) staining of larvae at 8 dpf. C1′ and C2′ are the enlarged images of the teeth region (n = 30 embryos per group). (**D**) MicroCT scans of adult zebrafish at 10 mpf. The hypurals in the red boxed area are used for analysis in E. (**E**) Summary of BV/TV for the bones in the boxed area in D (n = 3 zebrafish per group); mean grey-level intensities of the boxed area in D. (**F**) AR staining of adult zebrafish at 9 mpf. F1′–F3′″ are higher magnifications of boxed area in F1–F3. Mouths (F′), vertebral columns (F″) and caudal fins (F′″). The black arrows in F1′–F3′ point to the lower jaw; the black arrowheads in F2″-F3″ point to the neural arches; the blue arrowheads in F1′″–F3′″ point to hypurals 3–5 (**H3**–**5**); The blue arrows point to H1,2; N = 3 zebrafish per group. WT, wild type; HET, *mafbb^d^*^11*/+*^; HOM, *mafbb^d^*^11*/d*11^; BV, bone volume; TV, total tissue volume. Results in B and E are expressed as mean ± SEM, (* *p* < 0.05, **** *p* < 0.0001, *t* test).

To explore whether *mafbb* mutation causes any bone development defects, we performed alcian blue (AB) staining on 5 dpf embryos to visualize the cartilages of the pharyngeal skeleton [42]. The results showed that Meckel’s cartilage (mk) in 15% of *mafbb^+/−^* embryos is bent ventrally compared to WT (Figure 3A). In 75% of *mafbb^−/−^* embryos, mk, basihyal (bh), and ceratohyal (ch) are all bent ventrally (Figure 3A). In a small portion (5%) of *mafbb^−/−^* embryos, only three ceratobranchial pairs (cb) were clearly stained instead of 5 as seen in WT (Figure 3A3). We measured the ceratohyal angel (CHA) to quantify the cartiliage abnormality [43]. The CHA widens from 60° to around 100° (Figure 3B). Hence, *mafbb* mutation leads to an abnormal arrangement of pharyngeal skeleton. We used Alizarin red (AR) to stain for the bone mineralization in the larvae at 8 dpf. Although *mafbb^+/−^* embryos do not show changes in AR staining of bone and teeth compared to WT (data not shown), the *mafbb^−/−^* embryos demonstrate a strong reduction in the mineralization, and 10% of the embryos have a reduced number of mineralized teeth (Figure 3C).

To evaluate the bone formation at later stages, we stained 9 mpf adults with AR. The overall bone structure in the majority of *mafbb^+/−^* fish was comparable to WT, except for the lack of small holes frequently seen in hypural 1 and 2 next to the caudal fin in WT (Figure 3F). About half *mafbb^−/−^* zebrafish displayed the protruding lower jaw, curved spine and the corresponding abnormal bone structure. The shape of haemal and neural arches are more irregular and the hypurals are frequently fused in *mafbb^−/−^* (Figure 3F). Thus, zebrafish *mafbb* deficiency leads to defective cartilage and skeleton development. We further used micro-computed tomography(microCT) to examine the bone quality in adult fish [33,34]. As bone volume fraction (BV/TV, bone volume/total tissue volume) is a useful parameter for osteoporotic changes [33], we evaluated BV/TV for the hypural bones (Figure 3D). BV/TV is decreased in *mafbb^−/−^* compared to WT (Figure 3E). Moreover, the mean grey-level intensity in the hypural bones, as assessed by microCT [44], is also decreased in *mafbb^−/−^* compared to WT. These results are consistent with the phenotypes involved in osteoporosis, supporting the enhanced activation of osteoclast cells in the *mafbb^−/−^* mutants.

### 3.4. Macrophage Differentiation Is Altered in mafbb Mutants

**Figure 4:**
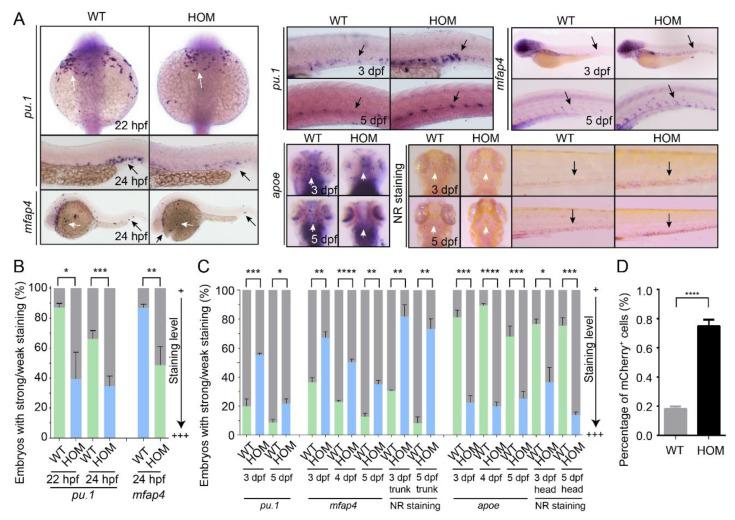
Macrophage development in WT and *mafbb* mutant embryos. (**A**) WISH of *pu.1*, *mfap4* and *apoe* in embryos at 22–24 hpf. WISH of *pu.1*, *mfap4* and *apoe*, neutral red (NR) staining in embryos at 3–5 dpf. The white arrows point to the rostral blood island and yolk sac, the black arrows point the ventral tail region. (**B**) Quantification of WISH results in embryos at 22–24 hpf (n = 50–100 embryos per group). (**C**) Quantification of WISH and NR staining in embryos at 3–5 dpf (n = 50–100 embryos per group). (**D**) Quantification of mCherry^+^ macrophage cells from Tg(*mpeg1*:mCherry) embryos at 5 dpf by flow cytometry. WT, wild type; HOM, *mafbb^d^*^11*/d*11^. Results in B-D are expressed as mean ± SEM, (* *p* < 0.05, ** *p* < 0.01, *** *p* < 0.001, **** *p* < 0.0001, *t* test).

Osteoclast cells are developed from the monocyte/macrophage precursors through a multistep process. While repressing osteoclastogenesis, MafB might enhance macrophage differentiation [6]. Similar to mammalian systems, zebrafish macrophage generation occurs in distinct waves during primitive and definitive hematopoiesis. The Ets transcription factor PU.1/Spi-1 is a master regulator of myeloid cell development. It is critical for macrophage differentiation, and also critical for the induction of *NFATc1*, *Ctsk*, and *TRAcP* during osteoclast differentiation [45]. We first examined the primitive macrophage development in *mafbb* mutants. The expression of *pu.1* and the macrophage specific marker *mfap4* in the rostral blood island and the ventral tail region are both significantly decreased in *mafbb^−/−^* mutant embryos compared to WT from 22–24 hpf (Figure 4A,B).

We then stained the embryos with *pu.1* and *mfap4* at later stages for definitive macrophage development. Surprisingly, *pu.1* and *mfap4* expression in the ventral trunk region (the circulating macrophages) in *mafbb^−/−^* is increased compared to WT at 3 and 5 dpf (Figure 4A,C). Similarly, neutral red (NR)-stained macrophages were increased in the tail of *mafbb* mutants. However, NR-stained macrophages in the brain (microglia) were significantly decreased in the mutants. A microglia specific marker *apoe* is also strongly reduced in *mafbb^−/−^* (Figure 4B,C). It is known that the embryonic microglia uniquely derive from primitive macrophages [46]. The reduced microglia development supports the reduction of primitive macrophages. To further characterize the macrophage development, we used Tg(*mpeg1*:mCherry) and performed flow cytometry to analyze the total number of mCherry^+^-macrophages in different embryos. At 5 dpf, the percentage of mCherry^+^ macrophages in *mafbb^−/−^* was significantly increased in comparison to WT (0.75 ± 0.02%, vs. 0.18± 0.01%, Figure 4D). Thus, *mafbb* mutants lead to expanded definitive macrophage differentiation and inhibit the primitive macrophage development.

### 3.5. Expansion of Definitive Myelopoiesis in mafbb Mutants

**Figure 5:**
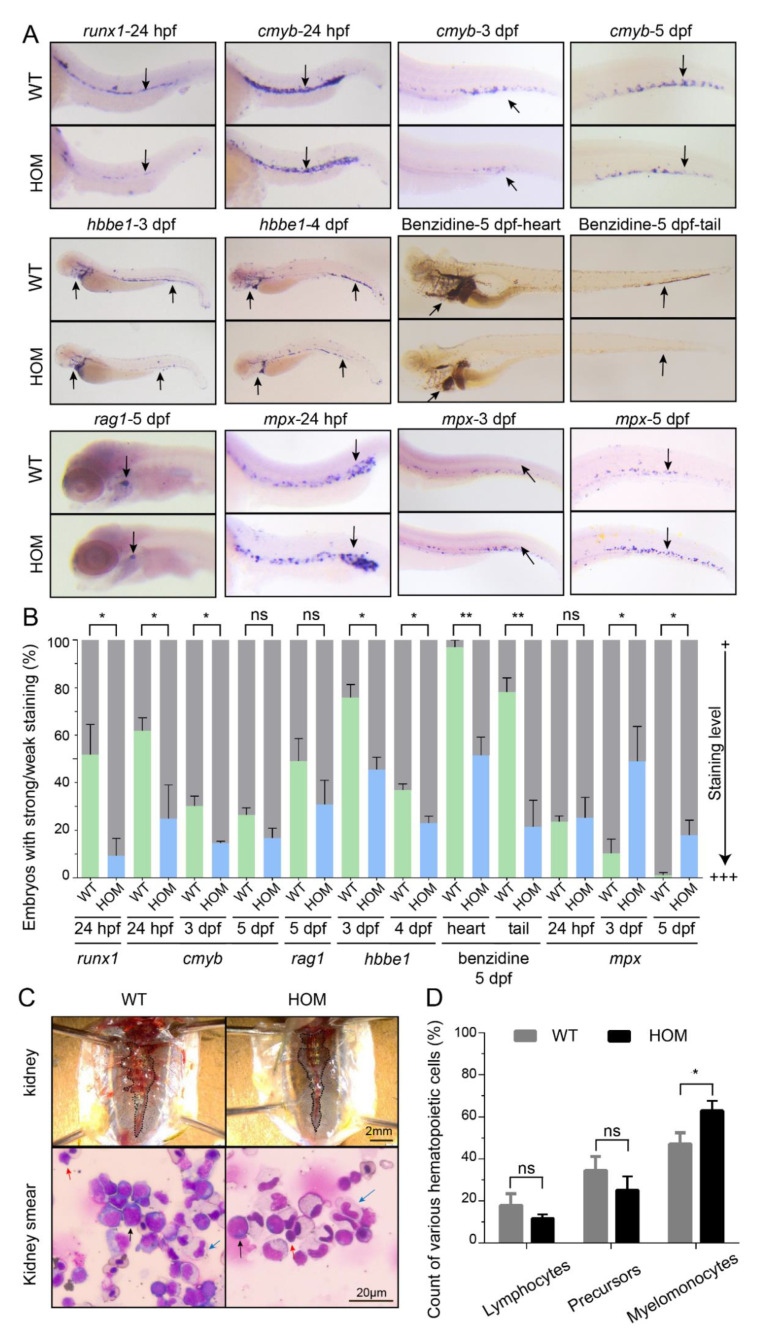
Expansion of definitive myeloid lineages in *mafbb* mutants. (**A**) WISH of *runx1*, *cmyb*, *mpx*, *rag1* and *hbbe1*, and benzidine staining of embryos during the early development stages. (**B**) Quantification of WISH and benzidine staining results in embryos (n = 50–100 embryos per group). (**C**) Images of the kidney in adult zebrafish. May-Grunwald and Giemsa (MGG) staining of the whole kidney marrow (WKM) blood smears from zebrafish at 9-month-old. Myelomonocytes (blue arrows); lymphocytes (red arrows); precursors (black arrows). (**D**) Summary of various hematopoietic cells from adult WKM. 3 zebrafishes and about 2000 cells in total were analyzed for each group. WT, wild type; HOM, *mafbb^d^*^11*/d*11^. Results in B and D are expressed as mean ± SEM, (* *p* < 0.05, ** *p* < 0.01, *t* test, ns, not significant).

Previous studies suggest that MafB might be involved in the development of multiple hematopoietic lineages in addition to macrophages [47]. To check whether lack of *mafbb* leads to preferential hematopoietic lineage differentiation, we examined the hematopoiesis in *mafbb^−/−^* by analyzing the development of various blood cells at different stages (Figure 5A,B). The results showed that the hematopoietic stem and progenitor cells (HSPCs), as stained by *runx1* and *cmyb*, were decreased in *mafbb^−/−^* at 24 hpf and 3 dpf, and were slightly decreased by 5 dpf. *rag1^+^*-T lymphocytes were slightly decreased at 5 dpf. The *hbbe1^+^* and benzidine stained erythrocytes are also decreased in *mafbb^−/−^* from 3–5 dpf. In contrast, *mpx* stained neutrophils were significantly increased in *mafbb^−/−^* compared to WT (Figure 5A,B), similar to the increased staining of myeloid progenitors and macrophages in *mafbb^−/−^* mutant during the definitive hematopoiesis. We then examined *mpx* expression in the primitive neutrophils, but did not see visible changes in *mafbb^−/−^* compared to WT (Figure 5A,B).

To study the myeloid lineages in adult zebrafish, we analyzed the hematopoietic cells from the whole kidney marrow (WKM) via cytological assay. The overall kidney morphology between WT and *mafbb^−/−^* are similar (Figure 5C,D). For the hematopoietic cells, the population of lymphoid cells, and of the precursors were all slightly decreased in *mafbb^−/−^*, but the population of myelomonocytes was expanded in *mafbb^−/−^*, consistent with the phenotypes during embryogenesis (Figure 5C,D). Thus, *mafbb* loss of function leads to selective expansion of definitive myelopoiesis including macrophages and neutrophils, and a small reduction of lymphoid and erythroid differentiation.

### 3.6. MCTO Mutant MAFB Does Not Rescue Osteolysis in mafbb^−/−^ Embryos

**Figure 6:**
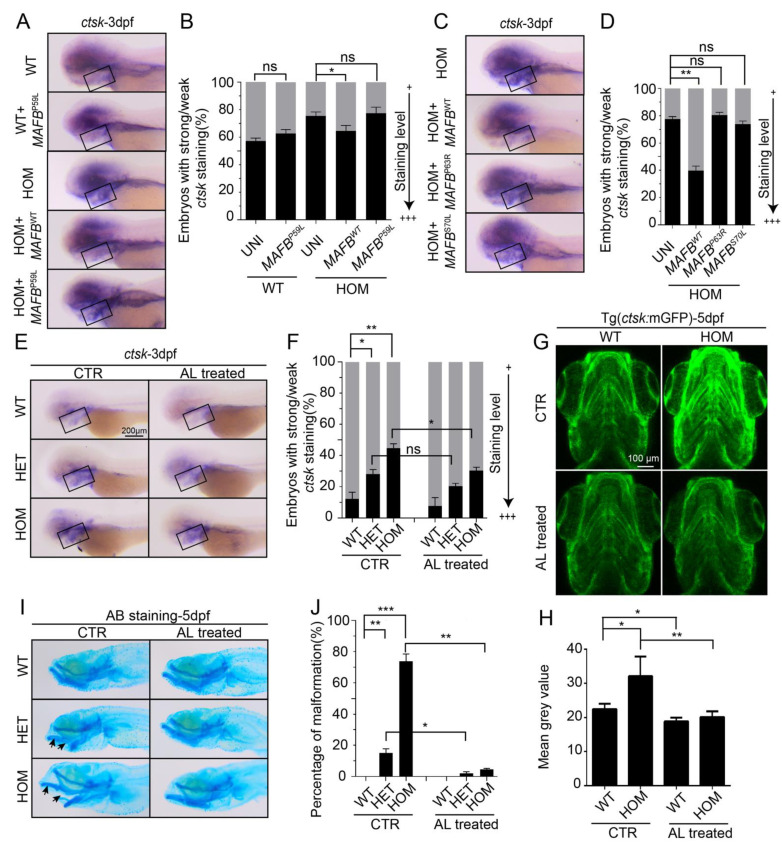
Rescue of *mafbb*^−/−^ embryos by WT and MCTO mutant MAFB and alendronate. (**A**) WISH of *ctsk* expression at 3 dpf in WT uninjected embryos, WT embryos injected with human P59L mutant *MAFB* mRNA, HOM uninjected embryos, and HOM embryos injected with human WT or P59L mutant *MAFB* mRNA. (**B**) Quantification of the *ctsk* expression in A. (**C**) WISH of *ctsk* expression at 3 dpf in HOM uninjected embryos, HOM embryos injected with human WT, P63R or S70L *MAFB* mRNA. (**D**) Quantification of the *ctsk* expression in C. (**E**) WISH of *ctsk* expression at 3 dpf in untreated and alendronate (AL)-treated embryos. (**F**) Quantification of the *ctsk* expression at 3 dpf in E. (**G**) Fluorescent images of Tg(*ctsk*:mGFP) at 5 dpf in WT and HOM embryos (the ventral view of head). (**H**) Mean grey value of fluorescence intensity of the images in G (n = 5 zebrafish per group). (**I**) AB staining of cartilages at 5 dpf in untreated and AL-treated embryos. Arrows indicate the lower jaw protrudes. (**J**) Quantification of AB staining in I. N = 50–100 embryos per group for A–F and I–J. WT, wild type; HET, *mafbb^d^*^11*/+*^; HOM, *mafbb^d^*^11*/d*11^; AL, alendronate. Results in B, D, F, J and H are expressed as mean ± SEM, (* *p* < 0.05, ** *p* < 0.01, *** *p* < 0.001, *t* test, ns, not significant).

MCTO individuals carry heterozygous missense mutations in MAFB with a dominant-inheritance pattern [14,15,16]. Whether the mutation in MCTO is haploinsufficiency or a dominant negative effect remains unclear. *mafbb^−/−^* zebrafish is a null mutant and has increased osteoclastogenesis, and Mafbb protein has 52% amino acid sequence identity with human MafB. We thus used this as a model to determine whether MCTO mutations could rescue the defects. The *mafbb^−/−^* embryos were injected with either WT or P59L mutated human *MAFB* mRNA. The WT *MAFB* mRNA reduced the overproduced *ctsk^+^*-cells, while the P59L *MAFB* mRNA did not (Figure 6A,B). Similarly, the P63R and S70L *MAFB* mRNA were not able to reduce *ctsk* expression in *mafbb^−/−^* (Figure 6C,D). In addition, the P59L *MAFB* mRNA injection in WT embryos did not interfere with the *ctsk* expression (Figure 6A,B). These results support that the MAFB MCTO mutations are loss-of-function mutations, and the heterozygosity of MCTO patients might be haploinsufficiency.

Currently, there are no effective treatments for MCTO patients [48]. Bisphosphonates may be effective by interrupting the osteoclast activity and formation [49]. A previous report suggested that bisphosphonates may slow down the progression of bone destruction in MCTO [16]. We exposed WT, *mafbb^+/−^*, *mafbb^−/−^* embryos to alendronate (AL), a type of bisphosphonate, and then examined the osteoclastogenesis and the cartilage development by *ctsk* expression and AB staining after the treatment. AL treatment reduced the increased *ctsk*^+^ expression in *mafbb^−/−^* embryos at 3 and 5 dpf (Figure 6E–H). More importantly, the cartilage abnormalities such as the jaw protrude, in *mafbb^−/−^* mutants were almost completely recovered after 4-day treatment of AL (Figure 6I,J). Thus bisphosphonates are capable of inhibiting the excessive osteoclastogenesis in *mafbb* mutants. The *mafbb^−/−^* mutant is a valid model to understand the pathophysiology of MCTO, and aid in the identification of novel therapies.

## 4. Discussion

Osteoclasts are produced from monocyte/macrophage lineage through a differentiation process induced by M-CSF and RANKL [12]. In bone marrow-derived monocyte/macrophage lineage cells, knockdown of MafB enhances RANKL-mediated osteoclastogenesis, while overexpression of MafB decreased the process [7]. Thus, MafB negatively regulates RANKL-induced osteoclastogenesis in vitro. However, the MafB function in vivo remains unclear. Mice with a homozygous deletion in the *MafB* gene or transgenic mice containing MCTO mutation (*MafB^MCTO/MCTO^*) have been generated, but their osteoclast development and bone formation defects have not been demonstrated.

In our studies, zebrafish *MafB* homolog *mafbb* deletion leads to consistently increased osteoclastogenesis and the subsequent bone growth defects, supporting that *MafB* also inhibits osteoclast development in vivo. The inhibition of osteoclastogenesis by *mafbb* might be dosage-dependent, as the *mafbb* heterozygotes also manifested considerable osteoclast cell overgrowth and bone development problems. Given the presence of an additional *MafB* homolog, *mafba* in zebrafish, *mafbb* homozygous mutants might not represent a MafB knockout, but are probably relevant for the autosomal dominant MCTO. *mafba* could play a compensatory role in osteoclastogenesis, it will be important to evaluate the function of *mafba* and the combined function of *mafba* and *mafbb* in osteoclastogenesis and bone growth to better reveal the conserved roles of *MafB* across different species.

In MCTO patients, only heterozygous missense mutations in MAFB are observed, and the mutations have a dominant inheritance pattern. Whether the pathogenesis of MCTO involves haploinsufficiency or a dominant-negative effect remains unknown. MCTO mutations all lie within a short region of the transcriptional activation domain of MAFB (amino acids 54–71), and map to the phosphorylation sites that determine MAFB protein stability [50]. It was hypothesized that MCTO mutations increase the MAFB protein stability and the individual might have enhanced MAFB expression [50]. Using zebrafish *mafbb* null mutants, we demonstrated MAFB MCTO mutations failed to reduce the excessive osteoclastogenesis in the mutant embryos. This provides evidence that MCTO mutations resemble loss of functions. Our findings are consistent with the finding showing the reduced transactivation activity of the mutated MAFB proteins [50]. We also injected mutant MAFB mRNA in WT embryos, and did not see noticeable effects on *ctsk* staining. Thus, MCTO mutations are likely haploinsufficiency. In the future, analysis of osteoclast differentiation in zebrafish or mice containing the MCTO mutations will help elucidate the precise pathogenesis of MCTO osteolysis.

While repressing osteoclastogenesis, MafB might enhance macrophage differentiation. Indeed, overexpression of *MafB* induced macrophage differentiation in chick myeloblasts [4]. However, in *MafB*-deficient mice, the number of macrophages was not impaired [6]. It was also found that combined knockout of MafB and C-Maf increases self-renewal of macrophages [51]. These discordant results highlight that the complex functions MafB in macrophages. Our studies indicate that MafB likely affects macrophage development differently at different stages. During the early developmental stages, the primitive myeloid progenitors and macrophages are strongly decreased in *mafbb* mutants. In support of this, the embryonic microglia, which exclusively derive from primitive macrophages, remain decreased in *mafbb* mutants. To our surprise, starting from 3 dpf, *pu.1* expression in myeloid cells, *mfap4*^+^-, NR stained- and Tg(*mpeg1*:mCherry) labeled-macrophages (circulating macrophages) are all expanded in *mafbb* mutants. Thus, *mafbb* deficiency leads to increased differentiation of definitive macrophages. Further studies using conditional and/or inducible deletion of *MafB* will help to dissect *MafB* function in macrophages during different stages and in different populations.

In *mafbb* mutants, HSPCs, the lymphoid cells and erythroid cells are all decreased or slightly decreased. However, the myeloid cells, including neutrophils in addition to macrophages, are significantly expanded, and the myeloid expansion remains till the adult stage. Our findings are consistent with the results by Sarrazin et al. [52]. They demonstrate that MafB deficiency leads to activation of myeloid master regulator Pu.1 and enhanced myeloid commitment in hematopoietic stem cell (HSC) reconstitution. Considering that osteoclast cells also originate from myeloid cell lineage, our result support that *mafbb* restricts definitive myeloid differentiation in vivo.

## 5. Conclusions

Here, we report that zebrafish *mafbb* deficiency leads to enhanced osteoclastogenesis and bone growth defects resembling MCTO. In addition, loss of *mafbb* in zebrafish reduces the primitive macrophage development, but expands definitive macrophage and myeloid lineage differentiation. Using *mafbb* mutants, we further demonstrate that MCTO missense mutations are loss of function mutations, and the pathogenesis of MCTO is likely haploinsufficiency.

## Data Availability

Not applicable.

## Appendix A

**Table A1:** Nucleotide sequence of primers used for *mafbb* mutants genotyping.

Primer	Nucleotide Sequence 5′-3′
DET F	GCGACGACAAACAGGCTAAT
DET R	GGGTGTGCATGCATGAGATT

**Table A2:** Nucleotide sequence of primers used for the probe.

Primer	Nucleotide Sequence 5′-3′
*rank* F	AAAA TCTAGA TGGGACTTTGCTGCAGTAGA
*rank* R	AAAA GAATTC GCCGTGATGCTGAGATTGAG
*c**tsk* F	AAAA TCTAGA CTGGCTCACTCTCTGGACAA
*ctsk* R	AAAA GAATTC AGCTCTCACATGACGGGAAA

**Table A3:** qPCR primers.

Primer	Nucleotide Sequence 5′-3′
*ctsk* F	ACCCAAACTGCAACAAGG
*ctsk* R	TAGCCCTTCTTTCCCCAC
*fosab* F	GGAGCAAAGACCTCCAACAA
*fosab* R	TCTTGTTTCGTTCACGACGTA
*nfatc1* F	CCGAGAGCAACATGAGAGC
*nfatc1* R	AGCTCGATGTCTGAGTTACGC
*opg* F	GTGAGTGTGAGGAGGGCTTC
*opg* R	TGTCACTGTACGGCGTTCC
*acp5a* F	CCATGTAGGAAACGTCAAAGC
*acp5a* R	GAATGCGGAAGTTCATCTCAT
*ocstamp* F	TCAGGTGGTCCTTGGATTTC
*ocstamp* R	AATGGGTACTTTTGTTCCAACCT
